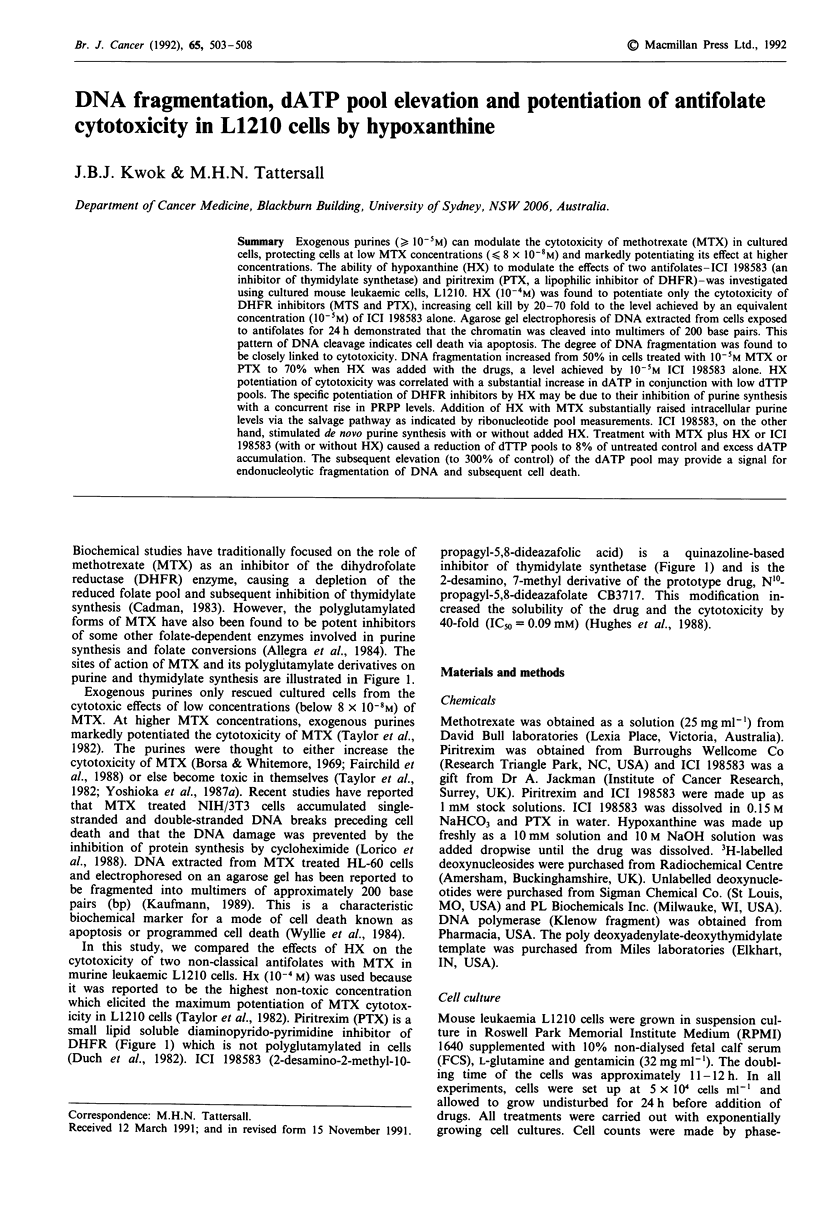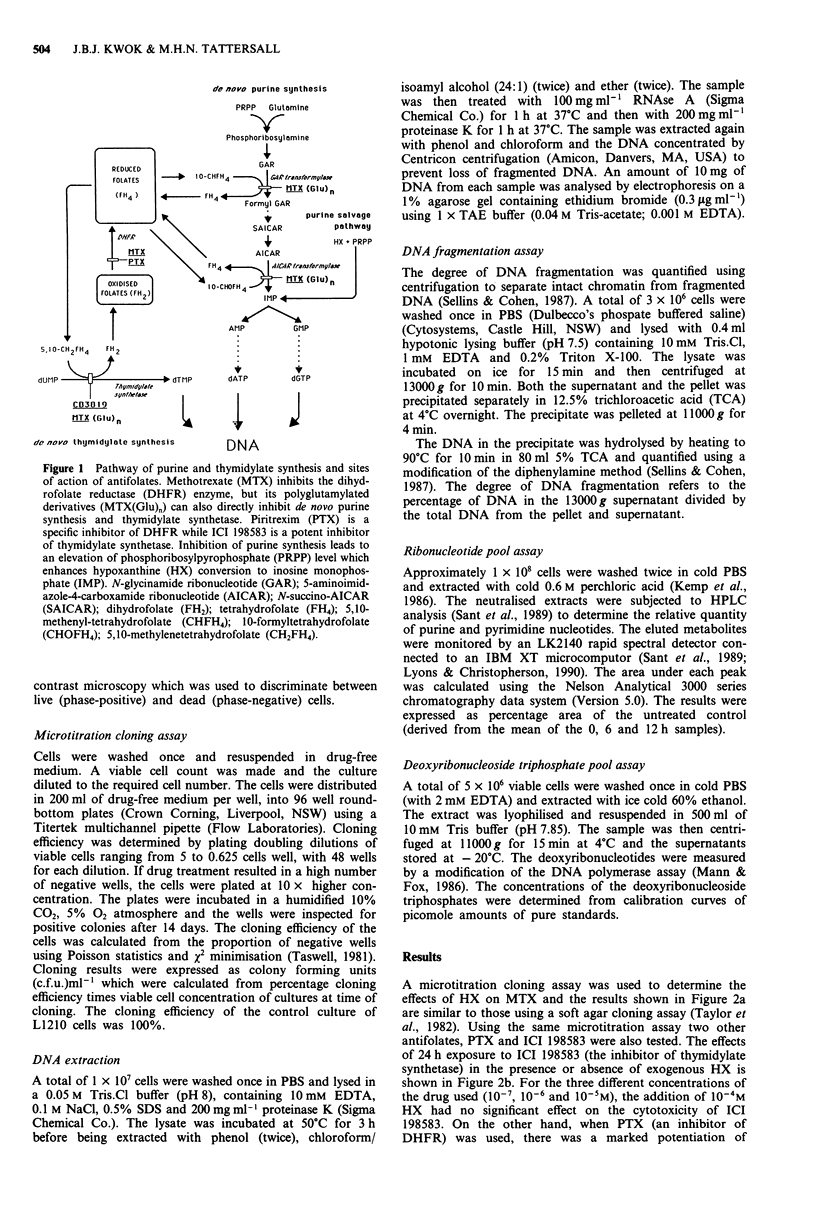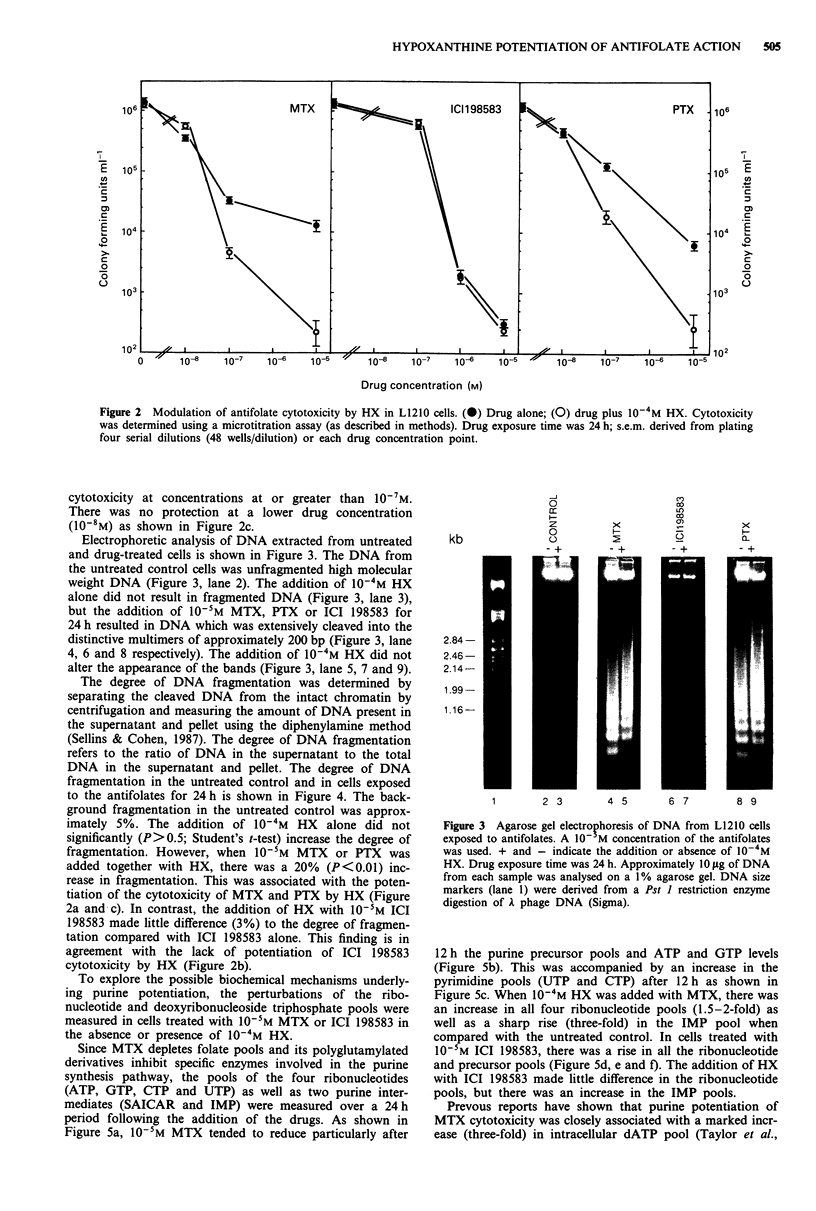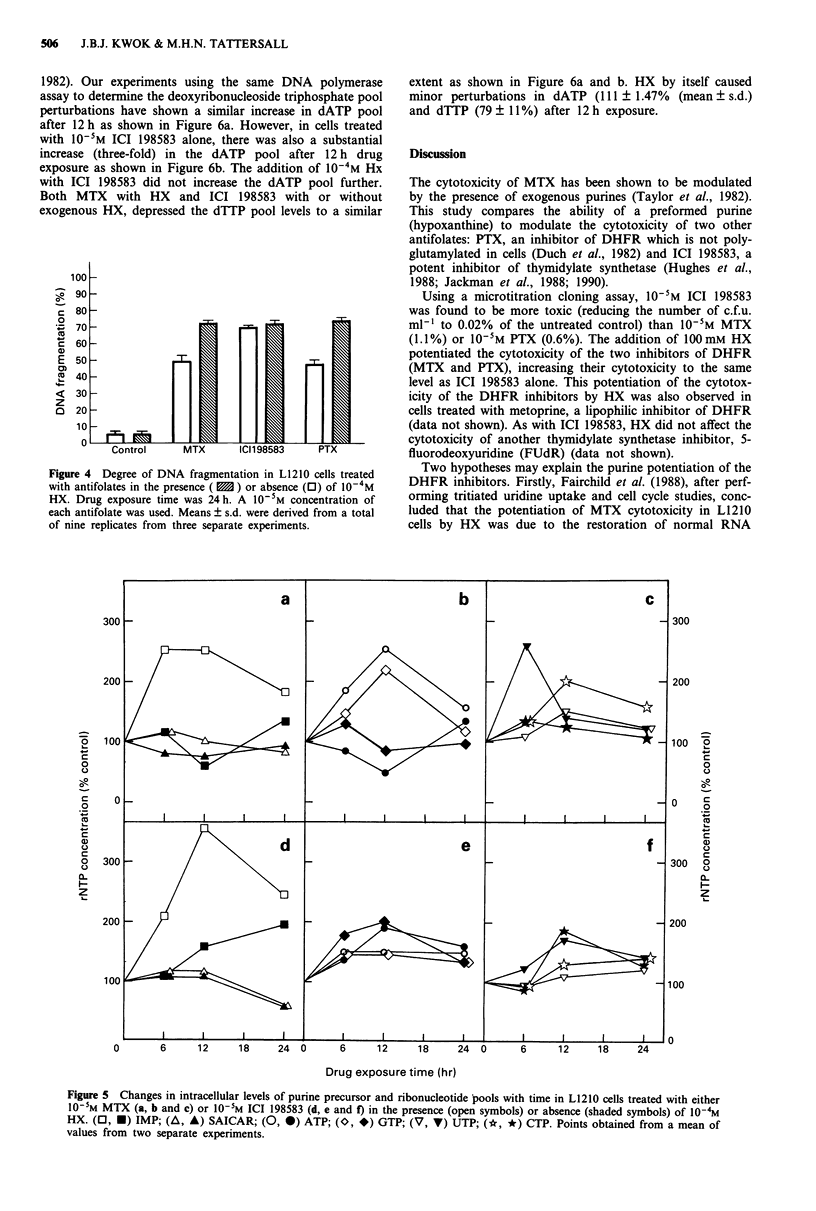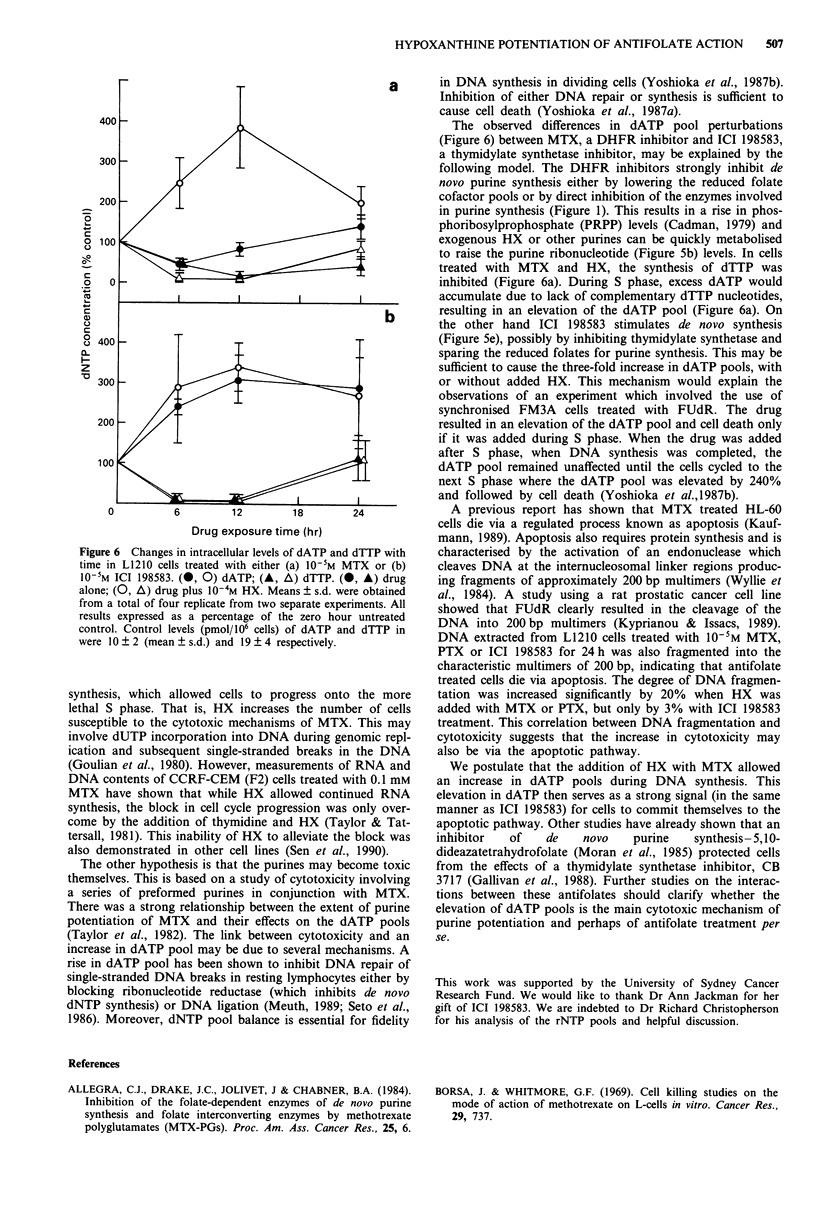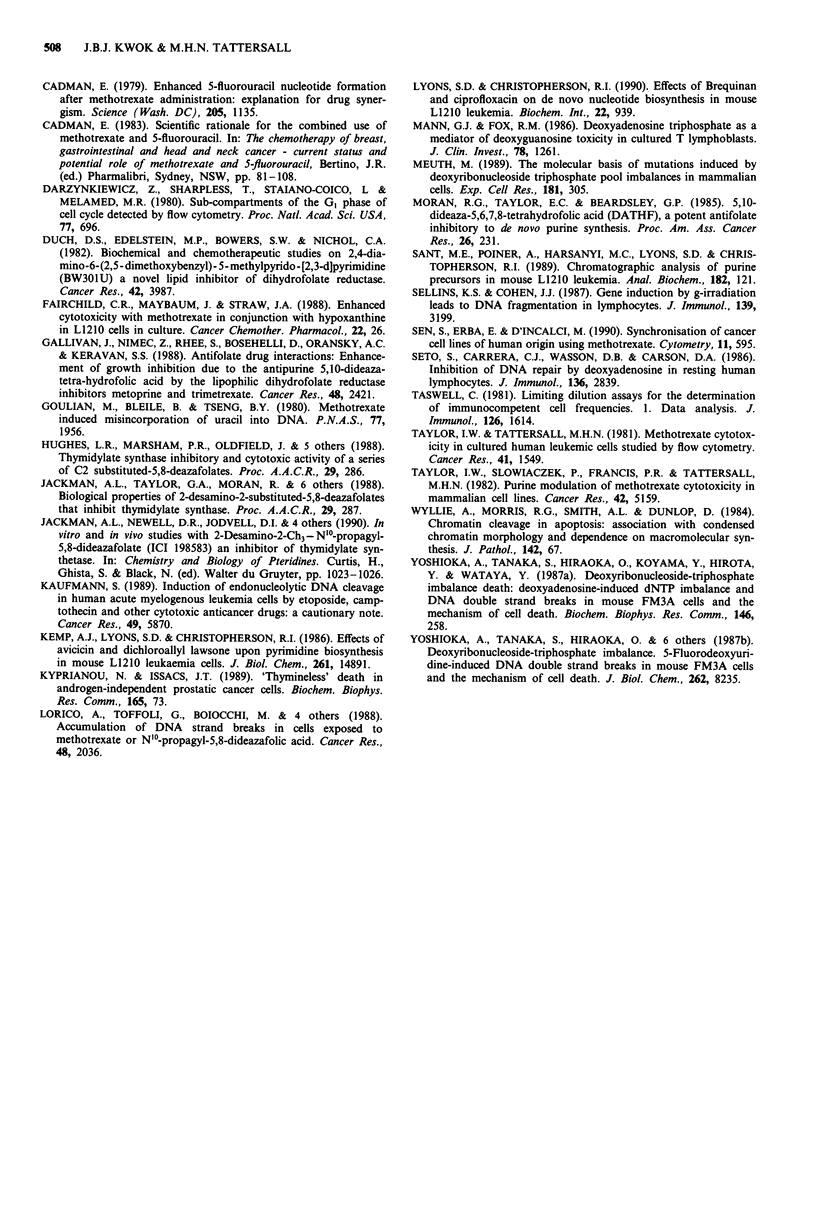# DNA fragmentation, dATP pool elevation and potentiation of antifolate cytotoxicity in L1210 cells by hypoxanthine.

**DOI:** 10.1038/bjc.1992.104

**Published:** 1992-04

**Authors:** J. B. Kwok, M. H. Tattersall

**Affiliations:** Department of Cancer Medicine, University of Sydney, NSW, Australia.

## Abstract

**Images:**


Br. J. Cancer (1992), 65, 503-508 © Macmillan Press Ltd., 1992

### DNA fragmentation, dATP pool elevation and potentiation of antifolate cytotoxicity in L1210 cells by hypoxanthine

J.B.J. Kwok & M.H.N. Tattersall

*Department of Cancer Medicine, Blackburn Building, University of Sydney, NSW 2006, Australia.*

**Summary** Exogenous purines ($\geqslant 10^{-5}$M) can modulate the cytotoxicity of methotrexate (MTX) in cultured cells, protecting cells at low MTX concentrations ($\leqslant 8 \times 10^{-8}$M) and markedly potentiating its effect at higher concentrations. The ability of hypoxanthine (HX) to modulate the effects of two antifolates-ICI 198583 (an inhibitor of thymidylate synthetase) and piritrexim (PTX, a lipophilic inhibitor of DHFR)-was investigated using cultured mouse leukaemic cells, L1210. HX ($10^{-4}$M) was found to potentiate only the cytotoxicity of DHFR inhibitors (MTS and PTX), increasing cell kill by 20-70 fold to the level achieved by an equivalent concentration ($10^{-5}$M) of ICI 198583 alone. Agarose gel electrophoresis of DNA extracted from cells exposed to antifolates for 24 h demonstrated that the chromatin was cleaved into multimers of 200 base pairs. This pattern of DNA cleavage indicates cell death via apoptosis. The degree of DNA fragmentation was found to be closely linked to cytotoxicity. DNA fragmentation increased from 50% in cells treated with $10^{-5}$M MTX or PTX to 70% when HX was added with the drugs, a level achieved by $10^{-5}$M ICI 198583 alone. HX potentiation of cytotoxicity was correlated with a substantial increase in dATP in conjunction with low dTTP pools. The specific potentiation of DHFR inhibitors by HX may be due to their inhibition of purine synthesis with a concurrent rise in PRPP levels. Addition of HX with MTX substantially raised intracellular purine levels via the salvage pathway as indicated by ribonucleotide pool measurements. ICI 198583, on the other hand, stimulated *de novo* purine synthesis with or without added HX. Treatment with MTX plus HX or ICI 198583 (with or without HX) caused a reduction of dTTP pools to 8% of untreated control and excess dATP accumulation. The subsequent elevation (to 300% of control) of the dATP pool may provide a signal for endonucleolytic fragmentation of DNA and subsequent cell death.

Biochemical studies have traditionally focused on the role of methotrexate (MTX) as an inhibitor of the dihydrofolate reductase (DHFR) enzyme, causing a depletion of the reduced folate pool and subsequent inhibition of thymidylate synthesis (Cadman, 1983). However, the polyglutamylated forms of MTX have also been found to be potent inhibitors of some other folate-dependent enzymes involved in purine synthesis and folate conversions (Allegra *et al.*, 1984). The sites of action of MTX and its polyglutamylate derivatives on purine and thymidylate synthesis are illustrated in Figure 1.

Exogenous purines only rescued cultured cells from the cytotoxic effects of low concentrations (below $8 \times 10^{-8}$M) of MTX. At higher MTX concentrations, exogenous purines markedly potentiated the cytotoxicity of MTX (Taylor *et al.*, 1982). The purines were thought to either increase the cytotoxicity of MTX (Borsa & Whitemore, 1969; Fairchild *et al.*, 1988) or else become toxic in themselves (Taylor *et al.*, 1982; Yoshioka *et al.*, 1987*a*). Recent studies have reported that MTX treated NIH/3T3 cells accumulated single-stranded and double-stranded DNA breaks preceding cell death and that the DNA damage was prevented by the inhibition of protein synthesis by cycloheximide (Lorico *et al.*, 1988). DNA extracted from MTX treated HL-60 cells and electrophoresed on an agarose gel has been reported to be fragmented into multimers of approximately 200 base pairs (bp) (Kaufmann, 1989). This is a characteristic biochemical marker for a mode of cell death known as apoptosis or programmed cell death (Wyllie *et al.*, 1984).

In this study, we compared the effects of HX on the cytotoxicity of two non-classical antifolates with MTX in murine leukaemic L1210 cells. Hx ($10^{-4}$ M) was used because it was reported to be the highest non-toxic concentration which elicited the maximum potentiation of MTX cytotoxicity in L1210 cells (Taylor *et al.*, 1982). Piritrexim (PTX) is a small lipid soluble diaminopyrido-pyrimidine inhibitor of DHFR (Figure 1) which is not polyglutamylated in cells (Duch *et al.*, 1982). ICI 198583 (2-desamino-2-methyl-10-propagyl-5,8-dideazafolic acid) is a quinazoline-based inhibitor of thymidylate synthetase (Figure 1) and is the 2-desamino, 7-methyl derivative of the prototype drug, $N^{10}$-propagyl-5,8-dideazafolate CB3717. This modification increased the solubility of the drug and the cytotoxicity by 40-fold ($IC_{50} = 0.09$ mM) (Hughes *et al.*, 1988).

Correspondence: M.H.N. Tattersall.
Received 12 March 1991; and in revised form 15 November 1991.

### Materials and methods

### Chemicals

Methotrexate was obtained as a solution (25 mg ml$^{-1}$) from David Bull laboratories (Lexia Place, Victoria, Australia). Piritrexim was obtained from Burroughs Wellcome Co (Research Triangle Park, NC, USA) and ICI 198583 was a gift from Dr A. Jackman (Institute of Cancer Research, Surrey, UK). Piritrexim and ICI 198583 were made up as 1 mM stock solutions. ICI 198583 was dissolved in 0.15 M $NaHCO_3$ and PTX in water. Hypoxanthine was made up freshly as a 10 mM solution and 10 M NaOH solution was added dropwise until the drug was dissolved. $^3$H-labelled deoxynucleosides were purchased from Radiochemical Centre (Amersham, Buckinghamshire, UK). Unlabelled deoxynucleotides were purchased from Sigman Chemical Co. (St Louis, MO, USA) and PL Biochemicals Inc. (Milwauke, WI, USA). DNA polymerase (Klenow fragment) was obtained from Pharmacia, USA. The poly deoxyadenylate-deoxythymidylate template was purchased from Miles laboratories (Elkhart, IN, USA).

### Cell culture

Mouse leukaemia L1210 cells were grown in suspension culture in Roswell Park Memorial Institute Medium (RPMI) 1640 supplemented with 10% non-dialysed fetal calf serum (FCS), L-glutamine and gentamicin (32 mg ml$^{-1}$). The doubling time of the cells was approximately 11-12 h. In all experiments, cells were set up at $5 \times 10^4$ cells ml$^{-1}$ and allowed to grow undisturbed for 24 h before addition of drugs. All treatments were carried out with exponentially growing cell cultures. Cell counts were made by phase-

**Figure 1** Pathway of purine and thymidylate synthesis and sites of action of antifolates. Methotrexate (MTX) inhibits the dihydrofolate reductase (DHFR) enzyme, but its polyglutamylated derivatives ($MTX(Glu)_n$) can also directly inhibit *de novo* purine synthesis and thymidylate synthetase. Piritrexim (PTX) is a specific inhibitor of DHFR while ICI 198583 is a potent inhibitor of thymidylate synthetase. Inhibition of purine synthesis leads to an elevation of phosphoribosylpyrophosphate (PRPP) level which enhances hypoxanthine (HX) conversion to inosine monophosphate (IMP). *N*-glycinamide ribonucleotide (GAR); 5-aminoimidazole-4-carboxamide ribonucleotide (AICAR); *N*-succino-AICAR (SAICAR); dihydrofolate ($FH_2$); tetrahydrofolate ($FH_4$); 5,10-methenyl-tetrahydrofolate ($CHFH_4$); 10-formyltetrahydrofolate ($CHOFH_4$); 5,10-methylenetetrahydrofolate ($CH_2FH_4$).

contrast microscopy which was used to discriminate between live (phase-positive) and dead (phase-negative) cells.

*Microtitration cloning assay*

Cells were washed once and resuspended in drug-free medium. A viable cell count was made and the culture diluted to the required cell number. The cells were distributed in 200 ml of drug-free medium per well, into 96 well round-bottom plates (Crown Corning, Liverpool, NSW) using a Titertek multichannel pipette (Flow Laboratories). Cloning efficiency was determined by plating doubling dilutions of viable cells ranging from 5 to 0.625 cells well, with 48 wells for each dilution. If drug treatment resulted in a high number of negative wells, the cells were plated at 10 × higher concentration. The plates were incubated in a humidified 10% $CO_2$, 5% $O_2$ atmosphere and the wells were inspected for positive colonies after 14 days. The cloning efficiency of the cells was calculated from the proportion of negative wells using Poisson statistics and $\chi^2$ minimisation (Taswell, 1981). Cloning results were expressed as colony forming units (c.f.u.)$ml^{-1}$ which were calculated from percentage cloning efficiency times viable cell concentration of cultures at time of cloning. The cloning efficiency of the control culture of L1210 cells was 100%.

*DNA extraction*

A total of $1 \times 10^7$ cells were washed once in PBS and lysed in a 0.05 M Tris.Cl buffer (pH 8), containing 10 mM EDTA, 0.1 M NaCl, 0.5% SDS and 200 mg $ml^{-1}$ proteinase K (Sigma Chemical Co.). The lysate was incubated at 50°C for 3 h before being extracted with phenol (twice), chloroform/isoamyl alcohol (24:1) (twice) and ether (twice). The sample was then treated with 100 mg $ml^{-1}$ RNAse A (Sigma Chemical Co.) for 1 h at 37°C and then with 200 mg $ml^{-1}$ proteinase K for 1 h at 37°C. The sample was extracted again with phenol and chloroform and the DNA concentrated by Centricon centrifugation (Amicon, Danvers, MA, USA) to prevent loss of fragmented DNA. An amount of 10 mg of DNA from each sample was analysed by electrophoresis on a 1% agarose gel containing ethidium bromide (0.3 $\mu g\ ml^{-1}$) using 1 × TAE buffer (0.04 M Tris-acetate; 0.001 M EDTA).

*DNA fragmentation assay*

The degree of DNA fragmentation was quantified using centrifugation to separate intact chromatin from fragmented DNA (Sellins & Cohen, 1987). A total of $3 \times 10^6$ cells were washed once in PBS (Dulbecco's phospate buffered saline) (Cytosystems, Castle Hill, NSW) and lysed with 0.4 ml hypotonic lysing buffer (pH 7.5) containing 10 mM Tris.Cl, 1 mM EDTA and 0.2% Triton X-100. The lysate was incubated on ice for 15 min and then centrifuged at 13000 *g* for 10 min. Both the supernatant and the pellet was precipitated separately in 12.5% trichloroacetic acid (TCA) at 4°C overnight. The precipitate was pelleted at 11000 *g* for 4 min.

The DNA in the precipitate was hydrolysed by heating to 90°C for 10 min in 80 ml 5% TCA and quantified using a modification of the diphenylamine method (Sellins & Cohen, 1987). The degree of DNA fragmentation refers to the percentage of DNA in the 13000 *g* supernatant divided by the total DNA from the pellet and supernatant.

*Ribonucleotide pool assay*

Approximately $1 \times 10^8$ cells were washed twice in cold PBS and extracted with cold 0.6 M perchloric acid (Kemp *et al.*, 1986). The neutralised extracts were subjected to HPLC analysis (Sant *et al.*, 1989) to determine the relative quantity of purine and pyrimidine nucleotides. The eluted metabolites were monitored by an LK2140 rapid spectral detector connected to an IBM XT microcomputor (Sant *et al.*, 1989; Lyons & Christopherson, 1990). The area under each peak was calculated using the Nelson Analytical 3000 series chromatography data system (Version 5.0). The results were expressed as percentage area of the untreated control (derived from the mean of the 0, 6 and 12 h samples).

*Deoxyribonucleoside triphosphate pool assay*

A total of $5 \times 10^6$ viable cells were washed once in cold PBS (with 2 mM EDTA) and extracted with ice cold 60% ethanol. The extract was lyophilised and resuspended in 500 ml of 10 mM Tris buffer (pH 7.85). The sample was then centrifuged at 11000 *g* for 15 min at 4°C and the supernatants stored at − 20°C. The deoxyribonucleotides were measured by a modification of the DNA polymerase assay (Mann & Fox, 1986). The concentrations of the deoxyribonucleoside triphosphates were determined from calibration curves of picomole amounts of pure standards.

### Results

A microtitration cloning assay was used to determine the effects of HX on MTX and the results shown in Figure 2a are similar to those using a soft agar cloning assay (Taylor *et al.*, 1982). Using the same microtitration assay two other antifolates, PTX and ICI 198583 were also tested. The effects of 24 h exposure to ICI 198583 (the inhibitor of thymidylate synthetase) in the presence or absence of exogenous HX is shown in Figure 2b. For the three different concentrations of the drug used ($10^{-7}$, $10^{-6}$ and $10^{-5}$M), the addition of $10^{-4}$M HX had no significant effect on the cytotoxicity of ICI 198583. On the other hand, when PTX (an inhibitor of DHFR) was used, there was a marked potentiation of

**Figure 2** Modulation of antifolate cytotoxicity by HX in L1210 cells. (●) Drug alone; (○) drug plus $10^{-4}$M HX. Cytotoxicity was determined using a microtitration assay (as described in methods). Drug exposure time was 24 h; s.e.m. derived from plating four serial dilutions (48 wells/dilution) or each drug concentration point.

cytotoxicity at concentrations at or greater than $10^{-7}$M. There was no protection at a lower drug concentration ($10^{-8}$M) as shown in Figure 2c.

Electrophoretic analysis of DNA extracted from untreated and drug-treated cells is shown in Figure 3. The DNA from the untreated control cells was unfragmented high molecular weight DNA (Figure 3, lane 2). The addition of $10^{-4}$M HX alone did not result in fragmented DNA (Figure 3, lane 3), but the addition of $10^{-5}$M MTX, PTX or ICI 198583 for 24 h resulted in DNA which was extensively cleaved into the distinctive multimers of approximately 200 bp (Figure 3, lane 4, 6 and 8 respectively). The addition of $10^{-4}$M HX did not alter the appearance of the bands (Figure 3, lane 5, 7 and 9).

The degree of DNA fragmentation was determined by separating the cleaved DNA from the intact chromatin by centrifugation and measuring the amount of DNA present in the supernatant and pellet using the diphenylamine method (Sellins & Cohen, 1987). The degree of DNA fragmentation refers to the ratio of DNA in the supernatant to the total DNA in the supernatant and pellet. The degree of DNA fragmentation in the untreated control and in cells exposed to the antifolates for 24 h is shown in Figure 4. The background fragmentation in the untreated control was approximately 5%. The addition of $10^{-4}$M HX alone did not significantly ($P>0.5$; Student's $t$-test) increase the degree of fragmentation. However, when $10^{-5}$M MTX or PTX was added together with HX, there was a 20% ($P<0.01$) increase in fragmentation. This was associated with the potentiation of the cytotoxicity of MTX and PTX by HX (Figure 2a and c). In contrast, the addition of HX with $10^{-5}$M ICI 198583 made little difference (3%) to the degree of fragmentation compared with ICI 198583 alone. This finding is in agreement with the lack of potentiation of ICI 198583 cytotoxicity by HX (Figure 2b).

To explore the possible biochemical mechanisms underlying purine potentiation, the perturbations of the ribonucleotide and deoxyribonucleoside triphosphate pools were measured in cells treated with $10^{-5}$M MTX or ICI 198583 in the absence or presence of $10^{-4}$M HX.

Since MTX depletes folate pools and its polyglutamylated derivatives inhibit specific enzymes involved in the purine synthesis pathway, the pools of the four ribonucleotides (ATP, GTP, CTP and UTP) as well as two purine intermediates (SAICAR and IMP) were measured over a 24 h period following the addition of the drugs. As shown in Figure 5a, $10^{-5}$M MTX tended to reduce particularly after

**Figure 3** Agarose gel electrophoresis of DNA from L1210 cells exposed to antifolates. A $10^{-5}$M concentration of the antifolates was used. + and − indicate the addition or absence of $10^{-4}$M HX. Drug exposure time was 24 h. Approximately 10 μg of DNA from each sample was analysed on a 1% agarose gel. DNA size markers (lane 1) were derived from a *Pst 1* restriction enzyme digestion of λ phage DNA (Sigma).

12 h the purine precursor pools and ATP and GTP levels (Figure 5b). This was accompanied by an increase in the pyrimidine pools (UTP and CTP) after 12 h as shown in Figure 5c. When $10^{-4}$M HX was added with MTX, there was an increase in all four ribonucleotide pools (1.5–2-fold) as well as a sharp rise (three-fold) in the IMP pool when compared with the untreated control. In cells treated with $10^{-5}$M ICI 198583, there was a rise in all the ribonucleotide and precursor pools (Figure 5d, e and f). The addition of HX with ICI 198583 made little difference in the ribonucleotide pools, but there was an increase in the IMP pools.

Prevous reports have shown that purine potentiation of MTX cytotoxicity was closely associated with a marked increase (three-fold) in intracellular dATP pool (Taylor *et al.*,

1982). Our experiments using the same DNA polymerase assay to determine the deoxyribonucleoside triphosphate pool perturbations have shown a similar increase in dATP pool after 12 h as shown in Figure 6a. However, in cells treated with $10^{-5}$M ICI 198583 alone, there was also a substantial increase (three-fold) in the dATP pool after 12 h drug exposure as shown in Figure 6b. The addition of $10^{-4}$M Hx with ICI 198583 did not increase the dATP pool further. Both MTX with HX and ICI 198583 with or without exogenous HX, depressed the dTTP pool levels to a similar extent as shown in Figure 6a and b. HX by itself caused minor perturbations in dATP (111 ± 1.47% (mean ± s.d.) and dTTP (79 ± 11%) after 12 h exposure.

**Figure 4** Degree of DNA fragmentation in L1210 cells treated with antifolates in the presence (▨) or absence (□) of $10^{-4}$M HX. Drug exposure time was 24 h. A $10^{-5}$M concentration of each antifolate was used. Means ± s.d. were derived from a total of nine replicates from three separate experiments.

### Discussion

The cytotoxicity of MTX has been shown to be modulated by the presence of exogenous purines (Taylor *et al.*, 1982). This study compares the ability of a preformed purine (hypoxanthine) to modulate the cytotoxicity of two other antifolates: PTX, an inhibitor of DHFR which is not polyglutamylated in cells (Duch *et al.*, 1982) and ICI 198583, a potent inhibitor of thymidylate synthetase (Hughes *et al.*, 1988; Jackman *et al.*, 1988; 1990).

Using a microtitration cloning assay, $10^{-5}$M ICI 198583 was found to be more toxic (reducing the number of c.f.u. $ml^{-1}$ to 0.02% of the untreated control) than $10^{-5}$M MTX (1.1%) or $10^{-5}$M PTX (0.6%). The addition of 100 mM HX potentiated the cytotoxicity of the two inhibitors of DHFR (MTX and PTX), increasing their cytotoxicity to the same level as ICI 198583 alone. This potentiation of the cytotoxicity of the DHFR inhibitors by HX was also observed in cells treated with metoprine, a lipophilic inhibitor of DHFR (data not shown). As with ICI 198583, HX did not affect the cytotoxicity of another thymidylate synthetase inhibitor, 5-fluorodeoxyuridine (FUdR) (data not shown).

Two hypotheses may explain the purine potentiation of the DHFR inhibitors. Firstly, Fairchild *et al.* (1988), after performing tritiated uridine uptake and cell cycle studies, concluded that the potentiation of MTX cytotoxicity in L1210 cells by HX was due to the restoration of normal RNA

**Figure 5** Changes in intracellular levels of purine precursor and ribonucleotide pools with time in L1210 cells treated with either $10^{-5}$M MTX (**a**, **b** and **c**) or $10^{-5}$M ICI 198583 (**d**, **e** and **f**) in the presence (open symbols) or absence (shaded symbols) of $10^{-4}$M HX. (□, ■) IMP; (△, ▲) SAICAR; (○, ●) ATP; (◇, ◆) GTP; (▽, ▼) UTP; (☆, ★) CTP. Points obtained from a mean of values from two separate experiments.

**Figure 6** Changes in intracellular levels of dATP and dTTP with time in L1210 cells treated with either (a) $10^{-5}$M MTX or (b) $10^{-5}$M ICI 198583. (●, ○) dATP; (▲, △) dTTP. (●, ▲) drug alone; (○, △) drug plus $10^{-4}$M HX. Means ± s.d. were obtained from a total of four replicate from two separate experiments. All results expressed as a percentage of the zero hour untreated control. Control levels (pmol/$10^6$ cells) of dATP and dTTP in were 10 ± 2 (mean ± s.d.) and 19 ± 4 respectively.

synthesis, which allowed cells to progress onto the more lethal S phase. That is, HX increases the number of cells susceptible to the cytotoxic mechanisms of MTX. This may involve dUTP incorporation into DNA during genomic replication and subsequent single-stranded breaks in the DNA (Goulian *et al.*, 1980). However, measurements of RNA and DNA contents of CCRF-CEM (F2) cells treated with 0.1 mM MTX have shown that while HX allowed continued RNA synthesis, the block in cell cycle progression was only overcome by the addition of thymidine and HX (Taylor & Tattersall, 1981). This inability of HX to alleviate the block was also demonstrated in other cell lines (Sen *et al.*, 1990).

The other hypothesis is that the purines may become toxic themselves. This is based on a study of cytotoxicity involving a series of preformed purines in conjunction with MTX. There was a strong relationship between the extent of purine potentiation of MTX and their effects on the dATP pools (Taylor *et al.*, 1982). The link between cytotoxicity and an increase in dATP pool may be due to several mechanisms. A rise in dATP pool has been shown to inhibit DNA repair of single-stranded DNA breaks in resting lymphocytes either by blocking ribonucleotide reductase (which inhibits *de novo* dNTP synthesis) or DNA ligation (Meuth, 1989; Seto *et al.*, 1986). Moreover, dNTP pool balance is essential for fidelity in DNA synthesis in dividing cells (Yoshioka *et al.*, 1987b). Inhibition of either DNA repair or synthesis is sufficient to cause cell death (Yoshioka *et al.*, 1987*a*).

The observed differences in dATP pool perturbations (Figure 6) between MTX, a DHFR inhibitor and ICI 198583, a thymidylate synthetase inhibitor, may be explained by the following model. The DHFR inhibitors strongly inhibit *de novo* purine synthesis either by lowering the reduced folate cofactor pools or by direct inhibition of the enzymes involved in purine synthesis (Figure 1). This results in a rise in phosphoribosylprophosphate (PRPP) levels (Cadman, 1979) and exogenous HX or other purines can be quickly metabolised to raise the purine ribonucleotide (Figure 5b) levels. In cells treated with MTX and HX, the synthesis of dTTP was inhibited (Figure 6a). During S phase, excess dATP would accumulate due to lack of complementary dTTP nucleotides, resulting in an elevation of the dATP pool (Figure 6a). On the other hand ICI 198583 stimulates *de novo* synthesis (Figure 5e), possibly by inhibiting thymidylate synthetase and sparing the reduced folates for purine synthesis. This may be sufficient to cause the three-fold increase in dATP pools, with or without added HX. This mechanism would explain the observations of an experiment which involved the use of synchronised FM3A cells treated with FUdR. The drug resulted in an elevation of the dATP pool and cell death only if it was added during S phase. When the drug was added after S phase, when DNA synthesis was completed, the dATP pool remained unaffected until the cells cycled to the next S phase where the dATP pool was elevated by 240% and followed by cell death (Yoshioka *et al.*,1987b).

A previous report has shown that MTX treated HL-60 cells die via a regulated process known as apoptosis (Kaufmann, 1989). Apoptosis also requires protein synthesis and is characterised by the activation of an endonuclease which cleaves DNA at the internucleosomal linker regions producing fragments of approximately 200 bp multimers (Wyllie *et al.*, 1984). A study using a rat prostatic cancer cell line showed that FUdR clearly resulted in the cleavage of the DNA into 200 bp multimers (Kyprianou & Issacs, 1989). DNA extracted from L1210 cells treated with $10^{-5}$M MTX, PTX or ICI 198583 for 24 h was also fragmented into the characteristic multimers of 200 bp, indicating that antifolate treated cells die via apoptosis. The degree of DNA fragmentation was increased significantly by 20% when HX was added with MTX or PTX, but only by 3% with ICI 198583 treatment. This correlation between DNA fragmentation and cytotoxicity suggests that the increase in cytotoxicity may also be via the apoptotic pathway.

We postulate that the addition of HX with MTX allowed an increase in dATP pools during DNA synthesis. This elevation in dATP then serves as a strong signal (in the same manner as ICI 198583) for cells to commit themselves to the apoptotic pathway. Other studies have already shown that an inhibitor of *de novo* purine synthesis-5,10-dideazatetrahydrofolate (Moran *et al.*, 1985) protected cells from the effects of a thymidylate synthetase inhibitor, CB 3717 (Gallivan *et al.*, 1988). Further studies on the interactions between these antifolates should clarify whether the elevation of dATP pools is the main cytotoxic mechanism of purine potentiation and perhaps of antifolate treatment *per se*.

This work was supported by the University of Sydney Cancer Research Fund. We would like to thank Dr Ann Jackman for her gift of ICI 198583. We are indebted to Dr Richard Christopherson for his analysis of the rNTP pools and helpful discussion.

### References

ALLEGRA, C.J., DRAKE, J.C., JOLIVET, J & CHABNER, B.A. (1984). Inhibition of the folate-dependent enzymes of *de novo* purine synthesis and folate interconverting enzymes by methotrexate polyglutamates (MTX-PGs). *Proc. Am. Ass. Cancer Res.*, **25**, 6.

BORSA, J. & WHITMORE, G.F. (1969). Cell killing studies on the mode of action of methotrexate on L-cells *in vitro*. *Cancer Res.*, **29**, 737.

CADMAN, E. (1979). Enhanced 5-fluorouracil nucleotide formation after methotrexate administration: explanation for drug synergism. *Science (Wash. DC)*, **205**, 1135.

CADMAN, E. (1983). Scientific rationale for the combined use of methotrexate and 5-fluorouracil. In: *The chemotherapy of breast, gastrointestinal and head and neck cancer - current status and potential role of methotrexate and 5-fluorouracil*, Bertino, J.R. (ed.) Pharmalibri, Sydney, NSW, pp. 81–108.

DARZYNKIEWICZ, Z., SHARPLESS, T., STAIANO-COICO, L & MELAMED, M.R. (1980). Sub-compartments of the $G_1$ phase of cell cycle detected by flow cytometry. *Proc. Natl. Acad. Sci. USA*, **77**, 696.

DUCH, D.S., EDELSTEIN, M.P., BOWERS, S.W. & NICHOL, C.A. (1982). Biochemical and chemotherapeutic studies on 2,4-diamino-6-(2,5-dimethoxybenzyl)-5-methylpyrido-[2,3-d]pyrimidine (BW301U) a novel lipid inhibitor of dihydrofolate reductase. *Cancer Res.*, **42**, 3987.

FAIRCHILD, C.R., MAYBAUM, J. & STRAW, J.A. (1988). Enhanced cytotoxicity with methotrexate in conjunction with hypoxanthine in L1210 cells in culture. *Cancer Chemother. Pharmacol.*, **22**, 26.

GALLIVAN, J., NIMEC, Z., RHEE, S., BOSEHELLI, D., ORANSKY, A.C. & KERAVAN, S.S. (1988). Antifolate drug interactions: Enhancement of growth inhibition due to the antipurine 5,10-dideaza-tetra-hydrofolic acid by the lipophilic dihydrofolate reductase inhibitors metoprine and trimetrexate. *Cancer Res.*, **48**, 2421.

GOULIAN, M., BLEILE, B. & TSENG, B.Y. (1980). Methotrexate induced misincorporation of uracil into DNA. *P.N.A.S.*, **77**, 1956.

HUGHES, L.R., MARSHAM, P.R., OLDFIELD, J. & 5 others (1988). Thymidylate synthase inhibitory and cytotoxic activity of a series of C2 substituted-5,8-deazafolates. *Proc. A.A.C.R.*, **29**, 286.

JACKMAN, A.L., TAYLOR, G.A., MORAN, R. & 6 others (1988). Biological properties of 2-desamino-2-substituted-5,8-deazafolates that inhibit thymidylate synthase. *Proc. A.A.C.R.*, **29**, 287.

JACKMAN, A.L., NEWELL, D.R., JODVELL, D.I. & 4 others (1990). *In vitro* and *in vivo* studies with 2-Desamino-2-$Ch_3-N^{10}$-propagyl-5,8-dideazafolate (ICI 198583) an inhibitor of thymidylate synthetase. In: *Chemistry and Biology of Pteridines*. Curtis, H., Ghista, S. & Black, N. (ed). Walter du Gruyter, pp. 1023–1026.

KAUFMANN, S. (1989). Induction of endonucleolytic DNA cleavage in human acute myelogenous leukemia cells by etoposide, camptothecin and other cytotoxic anticancer drugs: a cautionary note. *Cancer Res.*, **49**, 5870.

KEMP, A.J., LYONS, S.D. & CHRISTOPHERSON, R.I. (1986). Effects of avicicin and dichloroallyl lawsone upon pyrimidine biosynthesis in mouse L1210 leukaemia cells. *J. Biol. Chem.*, **261**, 14891.

KYPRIANOU, N. & ISSACS, J.T. (1989). 'Thymineless' death in androgen-independent prostatic cancer cells. *Biochem. Biophys. Res. Comm.*, **165**, 73.

LORICO, A., TOFFOLI, G., BOIOCCHI, M. & 4 others (1988). Accumulation of DNA strand breaks in cells exposed to methotrexate or $N^{10}$-propagyl-5,8-dideazafolic acid. *Cancer Res.*, **48**, 2036.

LYONS, S.D. & CHRISTOPHERSON, R.I. (1990). Effects of Brequinan and ciprofloxacin on de novo nucleotide biosynthesis in mouse L1210 leukemia. *Biochem. Int.*, **22**, 939.

MANN, G.J. & FOX, R.M. (1986). Deoxyadenosine triphosphate as a mediator of deoxyguanosine toxicity in cultured T lymphoblasts. *J. Clin. Invest.*, **78**, 1261.

MEUTH, M. (1989). The molecular basis of mutations induced by deoxyribonucleoside triphosphate pool imbalances in mammalian cells. *Exp. Cell Res.*, **181**, 305.

MORAN, R.G., TAYLOR, E.C. & BEARDSLEY, G.P. (1985). 5,10-dideaza-5,6,7,8-tetrahydrofolic acid (DATHF), a potent antifolate inhibitory to *de novo* purine synthesis. *Proc. Am. Ass. Cancer Res.*, **26**, 231.

SANT, M.E., POINER, A., HARSANYI, M.C., LYONS, S.D. & CHRISTOPHERSON, R.I. (1989). Chromatographic analysis of purine precursors in mouse L1210 leukemia. *Anal. Biochem.*, **182**, 121.

SELLINS, K.S. & COHEN, J.J. (1987). Gene induction by g-irradiation leads to DNA fragmentation in lymphocytes. *J. Immunol.*, **139**, 3199.

SEN, S., ERBA, E. & D'INCALCI, M. (1990). Synchronisation of cancer cell lines of human origin using methotrexate. *Cytometry*, **11**, 595.

SETO, S., CARRERA, C.J., WASSON, D.B. & CARSON, D.A. (1986). Inhibition of DNA repair by deoxyadenosine in resting human lymphocytes. *J. Immunol.*, **136**, 2839.

TASWELL, C. (1981). Limiting dilution assays for the determination of immunocompetent cell frequencies. 1. Data analysis. *J. Immunol.*, **126**, 1614.

TAYLOR, I.W. & TATTERSALL, M.H.N. (1981). Methotrexate cytotoxicity in cultured human leukemic cells studied by flow cytometry. *Cancer Res.*, **41**, 1549.

TAYLOR, I.W., SLOWIACZEK, P., FRANCIS, P.R. & TATTERSALL, M.H.N. (1982). Purine modulation of methotrexate cytotoxicity in mammalian cell lines. *Cancer Res.*, **42**, 5159.

WYLLIE, A., MORRIS, R.G., SMITH, A.L. & DUNLOP, D. (1984). Chromatin cleavage in apoptosis: association with condensed chromatin morphology and dependence on macromolecular synthesis. *J. Pathol.*, **142**, 67.

YOSHIOKA, A., TANAKA, S., HIRAOKA, O., KOYAMA, Y., HIROTA, Y. & WATAYA, Y. (1987a). Deoxyribonucleoside-triphosphate imbalance death: deoxyadenosine-induced dNTP imbalance and DNA double strand breaks in mouse FM3A cells and the mechanism of cell death. *Biochem. Biophys. Res. Comm.*, **146**, 258.

YOSHIOKA, A., TANAKA, S., HIRAOKA, O. & 6 others (1987b). Deoxyribonucleoside-triphosphate imbalance. 5-Fluorodeoxyuridine-induced DNA double strand breaks in mouse FM3A cells and the mechanism of cell death. *J. Biol. Chem.*, **262**, 8235.